# An Integrated Methodology for Bibliometric Analysis: A Case Study of Internet of Things in Healthcare Applications

**DOI:** 10.3390/s23010067

**Published:** 2022-12-21

**Authors:** Rahmat Ullah, Ikram Asghar, Mark G. Griffiths

**Affiliations:** CEMET, Faculty of Computing, Engineering and Science, University of South Wales, Pontypridd CF37 1DL, UK

**Keywords:** bibliometrics, internet of medical things, internet of things for medical devices, healthcare applications, bibliometric study

## Abstract

This paper presents an integrated and easy methodology for bibliometric analysis. The proposed methodology is evaluated on recent research activities to highlight the role of the Internet of Things in healthcare applications. Different tools are used for bibliometric studies to explore the breadth and depth of different research areas. However, these Methods consider only the Web of Science or Scopus data for bibliometric analysis. Furthermore, bibliometric analysis has not been fully utilised to examine the capabilities of the Internet of Things for medical devices and their applications. There is a need for an easy methodology to use for a single integrated analysis of data from many sources rather than just the Web of Science or Scopus. A few bibliometric studies merge the Web of Science and Scopus to conduct a single integrated piece of research. This paper presents a methodology that could be used for a single bibliometric analysis across multiple databases. Three freely available tools, Excel, Perish or Publish and the R package Bibliometrix, are used for the purpose. The proposed bibliometric methodology is evaluated for studies related to the Internet of Medical Things (IoMT) and its applications in healthcare settings. An inclusion/exclusion criterion is developed to explore relevant studies from the seven largest databases, including Scopus, Web of Science, IEEE, ACM digital library, PubMed, Science Direct and Google Scholar. The study focuses on factors such as the number of publications, citations per paper, collaborative research output, h-Index, primary research and healthcare application areas. Data for this study are collected from the seven largest academic databases for 2012 to 2022 related to IoMT and their applications in healthcare. The bibliometric data analysis generated different research themes within IoMT technologies and their applications in healthcare research. The study has also identified significant research areas in this field. The leading research countries and their contributions are another output from the data analysis. Finally, future research directions are proposed for researchers to explore this area in further detail.

## 1. Introduction

The recent trends, such as the growth in the elderly population, a shift in disease patterns, an increase in the proportion of women in the labour force, and a rising need for care, are among the factors driving the expansion of European home healthcare services [1]. However, these trends are also witnessed in other countries, and the demand for home healthcare is expected to rise globally. Home healthcare fills a void in the healthcare system by providing care at all three levels (primary, secondary, and tertiary) of pre-hospitalisation and post-hospitalisation. The current pandemic of COVID-19 has also brought much-needed attention to home healthcare [2]. Consequently, there has been a change in patient (consumer) behaviour regarding the consumption of healthcare services due to the increased risk of disease transmission. As a result, there is increased adoption of “Home Health” and “Telemedicine” services [3].

### 1.1. The Importance of IoT in Healthcare

While it is understood that technology cannot stop the population from ageing or eradicate chronic diseases at once, it can make healthcare more accessible and more cost-effective by equipping users with pocket-friendly medical facilities. A new paradigm, known as the Internet of Things (IoT), has extensive applicability in numerous areas, including healthcare [4]. The complete application of this paradigm in the healthcare service is of mutual benefit because it allows medical service providers to function proficiently and patients to obtain better treatment. With this technology-based healthcare method, unparalleled benefits could improve the quality and efficiency of medicines and the health of elderly patients.

### 1.2. Potential Advantages and Challenges

Although IoT in healthcare is a recent phenomenon, its advantages are already evident. The most important advantage of using IoT devices in healthcare is monitoring and reporting patient health conditions simultaneously. IoMT devices can also be helpful for patient data collection and analysis of this data. Patient health data analysis can then track their health and generate alerts if something unexpected occurs. IoMT devices not only enable remote health monitoring but can also help in providing remote medical assistance. In summary, IoMT can provide end-to-end connectivity between patients and their carers/doctors and can help reduce the cost of health monitoring and physical visits by medical professionals. The IoMT devices can also aid in further research on patient health, monitoring and telemedicine.

However, like any other technological field, IoT in healthcare comes with risks and challenges. The most significant risk can be data security and privacy [5]. Integrating different devices and protocols can pose multiple challenges to successfully implementing such systems in healthcare. With the passage of time and continuous use, such IoT systems are also prone to data overload and accuracy challenges [6]. The cost of purchasing/hiring IoT devices can also be challenging.

### 1.3. An Overview of the Related Studies

Recently, a bibliometric analysis of home health was performed using Scopus databases [4]. The objective of this study was to carry out a bibliometric analysis of Home Health with the Internet of Health Things (IoHT). A total of 1000 documents retrieved from Scopus were used for bibliometric analysis. The results have revealed that the key theme in the future could be a congruence of assisted living for older patients with health monitoring devices. The Internet of Things in Health trends is examined in [7] through bibliometrics and text mining. A total of 778 articles were extracted from the Web of Science from 1998 to 2016. Based on abstract text analysis, the papers were categorised into thirty clusters, resulting in eight trends of IoMT.

Similarly, a bibliometric study was performed to examine research publications in the smart home Internet of Things field [8]. A total of 2339 articles from the SCOPUS database released between 2015 and 2019 were used for analysis. A thorough study of Internet of Things (IoT) research from 1970 to 2019 is performed in [9]. The study adopts a bibliometric technique to examine the IoT trend by considering the publication year from its inception to the present. This study uncovers the publication patterns, significant prominent papers, publication locations, and several notable subjects. However, these studies are limited to the Scopus database only. A systematic mapping study explores the Internet of Things’ role in medicine [10]. This systematic mapping analysis evaluated studies published between 2000 and 2018 in key online scientific databases such as IEEE Xplore, Web of Science, Scopus, and PubMed. A total of 3678 papers were discovered, of which 89 were chosen based on inclusion/exclusion criteria. A bibliometric analysis of global research publications related to digital and mobile health was performed in [11]. The data were retrieved from Scopus due to its compatibility with the Bibliometrix R package. The analysis reveals a progressive increase in the publication of research articles on digital and mobile health until 2016, followed by a sudden surge in 2017.

There are various tools available to perform bibliometric analysis, including tools for data acquisition, performance analysis, science mapping, and visualisation. Currently, the Bibliometrix R package and its user-friendly web-based Biblioshiny platform have the most comprehensive set of techniques and are commonly used for bibliometric analysis [12]. A bibliometric analysis of gene editing using the R package on data retrieved from WoS is performed in [13]. Similarly, Scopus data were used to perform a bibliometric analysis of insurance literacy using the R package in [14]. The web-based interface of the R package, Biblioshiny, was used to perform bibliometric analysis on cross-cultural learning in [15]. In the study, WoS was used as a source of information, and the publication period was limited to 2002–2021. Microsoft Excel, VOSviewer, and Bibliometrix were used for statistical analysis of the publications in Scopus on the Omicron Variant of COVID-19 from 2020 to 2022 in [16]. In another study [7], BibExcel was used to perform a bibliometric analysis of IoMT. A total of 778 articles retrieved from the WoS were used to perform the analysis. A comprehensive overview of healthcare waste management research and its associated research themes and trends using VOSviewer was presented in [17]. It also identifies research gaps and proposes future avenues for research on sustainable healthcare waste management within the healthcare industry.

The Publish or Perish software has been previously used to retrieve data from Google Scholar (GS) for a bibliometric study on digital learning articles related to COVID-19 [18]. The paper also provides a step-by-step process of data retrieval from GS. Similarly, publish or perish software was used to assess the current state of IoT applications in irrigation [19]. The data were retrieved from the Scopus database and analysed using Publish or Perish and VOSviewer for citation and bibliometric map, respectively. The Scopus database data were used to examine the available scientific literature on the topic of Industry 4.0 [20]. Publish or Perish was used to include the collected data, while VOSviewer was utilised to visualise the data. Microsoft Excel and SPSS were used for data analysis. A similar work uses Scopus data to analyse Society 5.0 literature’s features, subjects, geographical distribution, keywords, and general themes [21]. Harzing’s Publish or Perish, and VOSviewer were used to analyse publication features, topics, and geographical distribution.

### 1.4. The Need for an Integrated Methodology

Bibliometric studies are used to evaluate research collaboration across scholars and institutions. This methodology provides a systematic, visible, and static depiction of research [22]. Bibliometric analysis is a well-established form of meta-analytical study [23] and a statistical technique that finds qualitative and quantitative changes in a particular research topic [4]. This research strategy has been utilised extensively to assess enormous quantities of publications in disciplines and domains as diverse as sustainability [24,25], healthcare [26,27,28] and green supply chain management [22,29], blockchain [30] and IoT [31,32]. Bibliometric analysis is a common and thorough technique for examining and interpreting vast quantities of scientific data [33]. It helps us examine a specific field’s evolutionary dynamics and offer insights into its emerging areas.

The current bibliometric studies retrieve papers from only Scopus or Web of Science (WoS) databases for analysis. A few researchers combine the two databases by undertaking a single analysis, although they do not describe the method. Recently, a technique of bibliometric analysis was proposed that merges Scopus and WoS databases [34]. A four-step procedure was presented to merge these two databases. The method was evaluated by conducting a bibliometric analysis of the sale force literature. The study assesses only two types of documents, articles and conference proceedings.

Similarly, a user-friendly method based on [34] is proposed to merge data obtained from Scopus and WoS [35]. The paper proposed a method to simplify some critical phases of merging datasets while performing bibliometric studies with the R package Bibliometric. A three-step, user-friendly method that does not require any coding skills or specialised software is provided. The proposed method can be used to perform a bibliometric study without compromising the integrity of the data. However, it is limited to only two databases.

Therefore, there is a need for an integrated methodology that considers all major databases for collecting data. This paper proposes an integrated methodology for bibliometric analysis to examine the state of the art of IoMT research. To our knowledge, this is the first extensive bibliometric analysis methodology that could be used to perform bibliometric analysis on data retrieved from multiple databases. The proposed bibliometric methodology will enable researchers new to a field to find leading articles based on citation counts, prolific authors, and research hotspots from multiple databases. The method is suitable for assessing the present state of a given field based on markers such as highly cited publications, researchers, journals, academic institutions, and countries. The proposed method could be used to perform an integrated bibliometric analysis in any research field without the use of coding or specialised tools.

### 1.5. Research Objectives

Traditionally, bibliometric studies cover a research domain from broader angles and provide an in-depth analysis of that research domain. Although there are various surveys, systematic mappings and bibliometric studies performed, for example, the Internet of Things research, there is no study conducted to explore the Internet of medical things as an emerging field. Therefore, this paper aims to aggregate knowledge from major research databases related to IoT use in healthcare and highlight essential research themes within this research domain. This aim is achieved by accomplishing the following research objectives.

1. The first objective of this research is to present an integrated methodology to perform bibliometric analysis based on data from multiple sources rather than just WoS and Scopus.
2. To describe the data collection, data cleaning and integration process of the proposed methodology in detail, along with the tools required.
3. To evaluate the proposed methodology by performing a bibliometric study on IoMT research and its applications in healthcare settings.
4. To use a systematic approach to identify the most influential scholars, their associations, their chosen keywords, and, most importantly, the connections between academic works [36].
5. To explore essential research themes and highlight open areas that need further research efforts.

## 2. Proposed Methodology

This section describes the details of the proposed methodology used for this study. The proposed methodology consists of three significant steps: data collection, integration, and cleaning.

### 2.1. Data Collection

For data collection, we have used Scopus, Web of Science, IEEE, ACM, PubMed, Science Direct, and GS. Search strings are used to extract relevant articles to download the relevant data. Scopus, PubMed, and Web of Science allow users to download the search results in various formats, including CSV, Excel, and Latex. The results of the search were downloaded and saved as .bib file. The advanced search feature of IEEE, Science Direct and GS was used. The advanced search allows users to specify more targeted search criteria before conducting a search. A simple tutorial for performing an advanced search for IEEE Xplore and Science Direct can be found in [37] and [38], respectively. The advanced search option on Google Scholar provides additional search filters and options that can help users narrow down their search results and find more relevant articles. These advanced search options include filters such as the publication date range, authors and source. A step-by-step process of retrieving data using an advanced GS search can be found in Appendix A. The results were exported in BibTeX format for IEEE and Science Direct.

The search string in these databases was limited to the article title, abstract, and keywords, except in GS, where the search string was limited to the document title only. This is because GS does not allow a search based on the abstract and keywords. For ACM digital library, a maximum of 50 papers’ data can be displayed at one time. These data were selected and exported as a .bib file. The data were downloaded in chunks of 50 papers and merged into one .bib file. GS, however, does not have any such feature. To retrieve GS results, the Publish or Perish software is used [39]. The Publish or Perish software does not allow downloading more than 1000 results. Therefore, the search string is divided into sub-strings and used to retrieve the relevant results. It is essential to ensure that the search string in GS and the Publish or Perish software retrieve the same results. The search strings used to retrieve the results can be found in Table 1.

The retrieved results using Publish or Perish will not contain citation information essential for citation, co-citation, and the most popular authors’ analyses. Publish or Perish allows users to inspect and modify the attributes of the selected data and search for citations. The GS citing references pane displays information regarding a citing reference search. When one or more results are chosen in a list of another search, citing information can be retrieved. For both sub-strings for GS, the citation information was retrieved using Publish or Perish. The retrieved data were then exported to an Excel file with headers. To use these data in Biblioshiny, it needs to be formatted before integrating with data from other databases. The keywords are separated by ‘,’ which needs to be replaced with a ‘;’. Similarly, the names of the tag are different. The tags are renamed according to the guidelines provided by Biblioshiny. The data need to be formatted according to the format exported from R.

### 2.2. Data Integration

In previous bibliometric studies, authors have used the Web of Science or Scopus databases separately due to their different output results. This work integrates data from various search engines, presenting a holistic view. This section describes the data integration process. There are two different ways to incorporate the data. The R package or Excel software can be used. We chose Excel for its various advantages, including its capability to retain uncommon tags (columns of data), which is of utmost importance for bibliometric analysis. The R package is an easy and more efficient way to integrate data into databases with similar tags. However, it discards the uncommon columns and merges the data with similar tags. The methodology for data integration using R can be found in Appendix B.

This study uses Biblioshiny and Excel to integrate the data from all seven databases. Biblioshiny is a web-based application embedded in the bibliometric package [40]. The functionality of Bibliometric is combined with the web interface using the Shiny packaged environment. The advantage of the Biblioshiny is that it allows non-coders to use the Bibliometric package. The data were imported from all databases to Biblioshiny and exported to Excel one by one. The format of the data can be found in Table 1. A step-by-step guide on how to merge the data exported from Biblioshiny and Perish and Publish (for GS) using Excel is provided in Appendix C.

### 2.3. Data Cleaning

Data cleaning is a necessary process to remove irrelevant and duplicate articles. In data integration, all the raw data were integrated without considering duplicate articles. The duplicate criteria were set to author, year, and title. These criteria ensured that different papers with the same title were not removed. Using R is just a line of code to remove duplicates, but it deletes all uncommon columns, resulting in data loss. In Excel, the data were cleaned using the following steps.

1. Remove any spaces in the merged file in the Author “AU” column.
2. Replace the Empty values for “SR” and “SR full” columns as Nil for GS Data.
3. Sort the data using the “PY” column in Excel and remove any documents before 2012 and after 2022 (there may be some early access articles).
4. Fully automated duplicate detection and merging are challenging and almost impossible, at least if accuracy/precision is valued. While there are ways to match records 1 to 1, such as with a DOI, different databases can have different degrees of completeness for items such as author names, etc. Human decision-making will always need to be involved in the selection and merging process. This is because of similar records, except one has information that the other does not. Some records could have different information for the same field but are otherwise identical. Therefore, the duplicates are removed using “TI”, AU and PY. The “Remove Duplicates” features in Excel (In the Data Tab) are used. This removed 668 using author, documents title and publication year. This will delete only those documents with the same data in all fields, such as Authors, title, and year but will not delete all duplicates. Furthermore, the TI and URL were used to remove the remaining duplicates.
5. To check if all the duplicates are removed, the Conditional Formatting (in the Home Tab), Highlight Cell rules -> Duplicate values are used. This will highlight all the duplicated titles. These duplicates then need to be merged. It is essential to ensure that these duplicates are not deleted but merged. The potential problem is merging multiple types of items, for example, the same article in a journal and a book chapter. Users need to change the type of one or more items into identical types and then merge the item to remove the duplicates. This is a time-consuming process, but it would make sure that the data are properly cleaned. In this study, the highlighted documents were compared against other available tags, such as year, type, etc., and eliminated accordingly. The duplicated function does not delete documents where author names are in a different order or where long names are used. These documents need to be removed manually. A total of 327 documents were removed.
6. Save the cleaned data in xlsx format.

## 3. Results and Discussion

### 3.1. Descriptive Statistics

A total of 5304 articles (including books, journals and conference papers) were used for bibliometric analysis. These documents were collected from 2365 different sources published between 2012 and 2022 (July 2022). The total number of authors is 14,488, according to the statistics provided by Biblioshiny [41]. An overview of the data used for bibliometric analysis can be found in Table 2.

**Figure 1:**
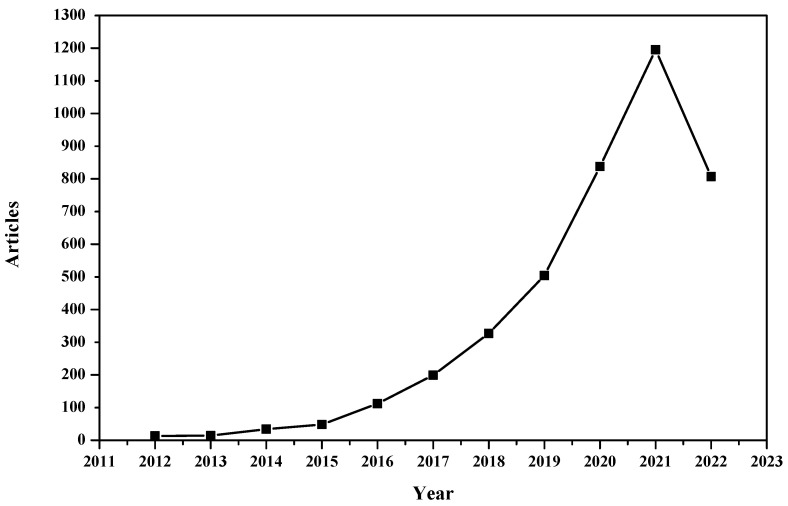
Annual Scientific Production.

The annual scientific production of articles can be found in Figure 1 and Table 3. The figure shows the compound annual growth rate that shows the geometric progression ratio over a time span. It can be observed that IoMT research is growing exponentially. It is essential to mention that the number of articles for 2022 is until July 2022 (data retrieval time).

The following table shows the annual production of articles, mean total citations per article, mean total citations per year and citation years.

**Figure 2:**
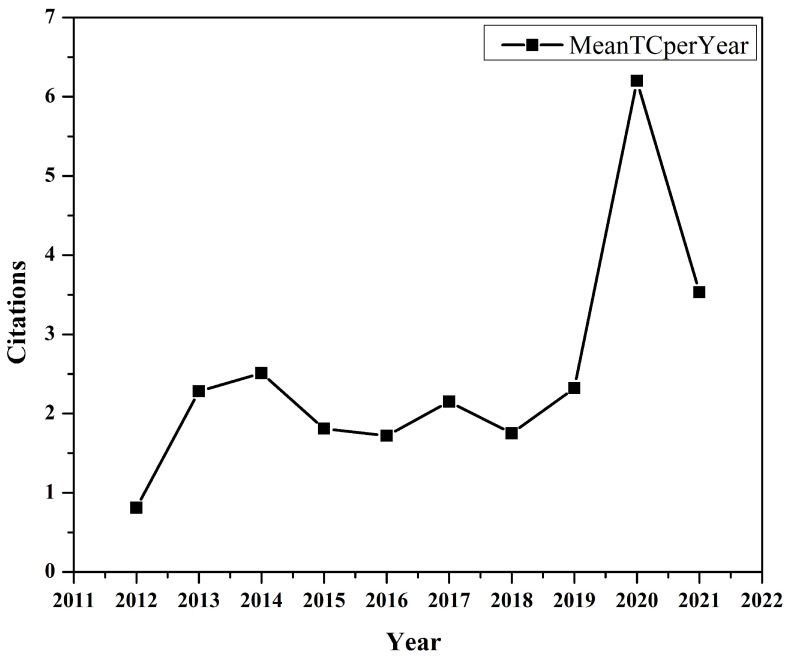
Average Citation Per Year.

The citation graph shown in Figure 2 indicates each year’s average paper citation in the dataset. In our collection, one or more articles published in 2020 got the highest number of citations per year. The graph shows that average citations were slightly higher in 2013 and 2014 compared to 1015-1018. A significant increase in the number of citations can be seen in 2020 and 2021, which shows that IoMT is an emerging topic for research.

### 3.2. Most Relevant Sources

**Figure 3:**
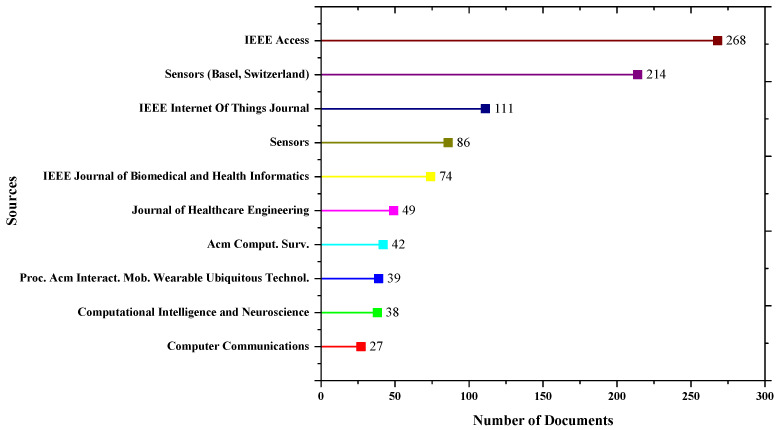
The Top 10 Most Relevant Sources publishing IoMT articles.

A source is a journal, conference, proceeding or book published in one or more sources in the bibliographic collection. A total of 2365 sources published research related to IoMT from 2012 to July 2022. More sources are due to GS data as GS search contains data from all sources, including presentations, white papers, etc. This also indicates that many sources are publishing IoMT-related research articles. The top 10 sources publishing IoMT research are shown in Figure 3 and Table 4. The figure shows the number of documents published in each source. The open access IEEE journal IEEE access has published 268 documents, followed by the Sensors journal.

Table 4 shows the *h*, *g* and *m* indexes for the top ten sources The Hirsch index (*h*-index) is the number of published articles by an author or journal (in this case), each of which has been cited at least *h* times in other articles. Let *f* be the function that assigns each publication *i* its citation count, and let *f* be sorted in decreasing order. The h-index is therefore expressed as [42]:

(1) $$h=\max_{i}\min\left(f\left(i\right),i\right)$$

The *g*-index, on the other hand, is an improvement to the *h*-index that measures the global citation performance of a set of articles. The set of articles is ranked in decreasing order of received citations. The g-index shows the most significant number that the top *g* articles received at least *g*${}^{2}$ citations.

(2) $$g^{2}\leq \sum_{i\leq g}c_{i}$$

The *m*-index is the ratio of *h* and *n* and can be calculated as:

(3) h/n

where *n* is the number of years since the first publication of an author or a journal.

**Figure 4:**
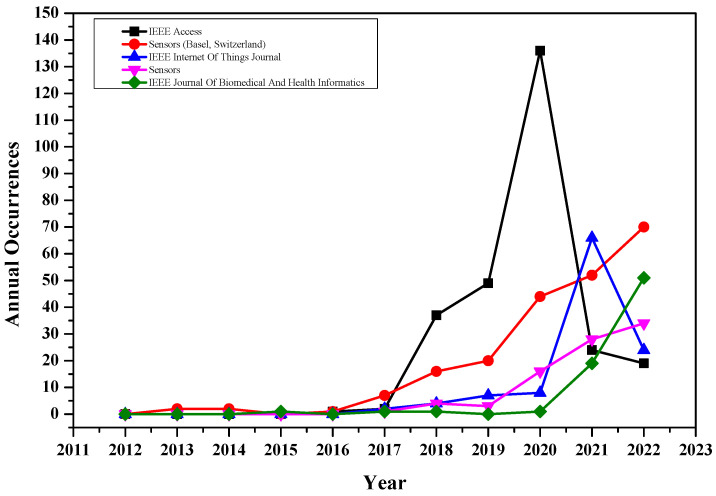
The number of publications per year.

Table 4 also shows the total citations for each source. Articles published in IEEE access are the most cited, followed by the IEEE Internet of Things journal. The data for total publications show that Sensors (MDPI) has been published more than twice compared to the IEEE Internet of Things journal. However, the citation for IEEE Internet of Things journal is significantly higher than Sensor’s journal. Contrarily, open-access journals are publishing more articles compared to subscription-based journals. The annual publication occurrences for the top five journals can be found in Figure 4. The IEEE access has published more articles in the past five years, followed by the Sensors (MDPI) Journal.

### 3.3. Influential Authors, Their Affiliations and Countries

**Figure 5:**
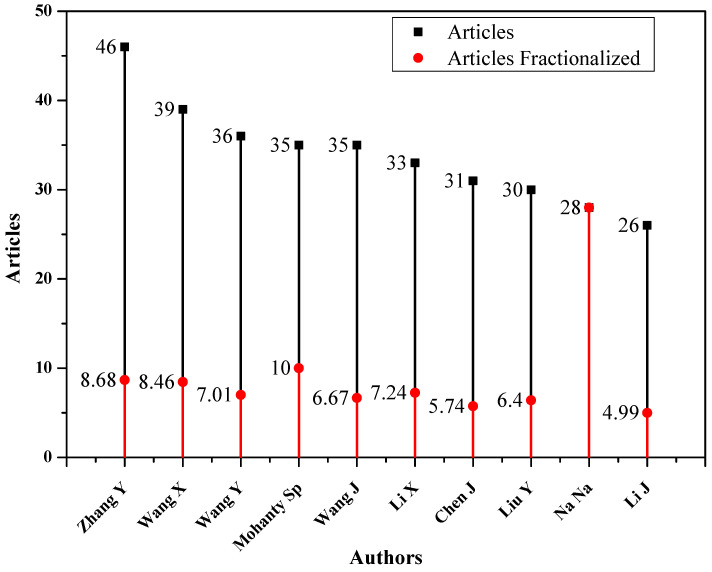
Most relevant and influential authors for IoMT research.

Knowing about the relevant groups and top authors is of utmost importance, especially for early career researchers. There are many ways to follow the relevant authors and get updates on their new research output. The GS provides email notifications of new articles for all the following authors. Another way is to follow the authors on various social platforms, such as ResearchGate or LinkedIn. The most relevant and influential authors regarding IoMT research can be found in Figure 5. The figure shows the total articles (denoted by black lines) and fractionalised articles (indicated by red lines).

The fractional authorship quantifies an author’s contributions to a published set of articles, assuming all co-authors contributed to each document equally. The fractional frequency is calculated as

(4) $$\;\mathrm{Frac}\;\mathrm{Freq}\;\left(AU_{j}\right)=\sum_{h\in AU_{j}}\frac{1}{numberofCoAuthors(h)}$$

where $AU_{j}$ denotes a set of articles co-authored by an author *j*, and *h* is the document included in $AU_{j}$.

**Figure 6:**
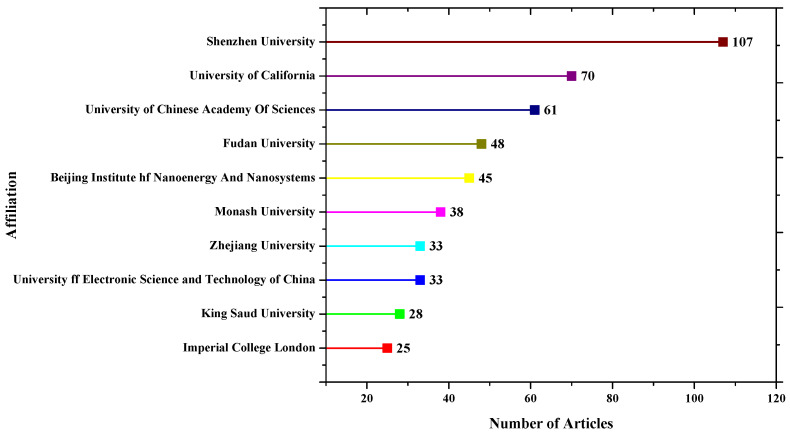
Most relevant affiliation.

In terms of most relevant affiliations based on the corresponding authors of articles, Shenzhen University has the most articles, followed by King Saud University, as shown in Figure 6.

**Figure 7:**
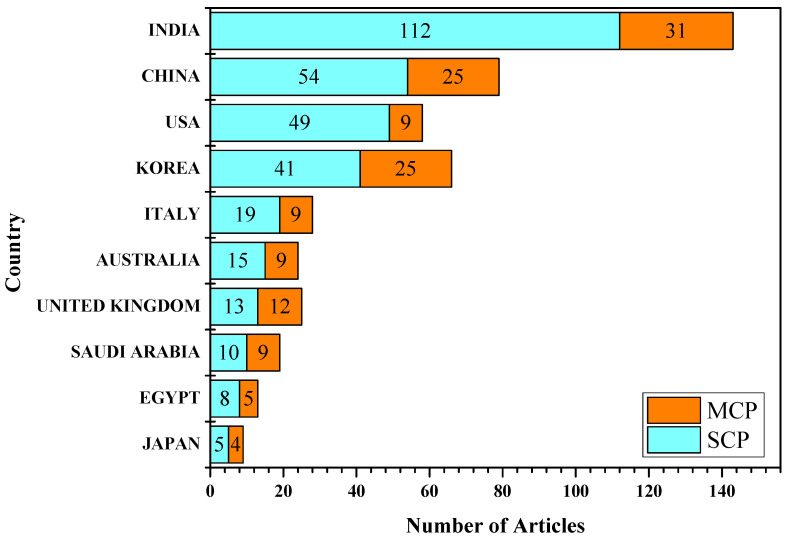
Number of articles based on Corresponding author, MCP: Multiple Countries Publications, SCP: Single Country Publications.

The number of publications where each article is assigned to a specific country based on the affiliation of the accompanying author is shown in Figure 7. In this instance, the overall number of articles correlates to the frequency per country. In addition, this analysis determines the fraction of publications in which at least one author is affiliated with a country other than that of the corresponding author. The index is known as Multiple Country Publications (MCP).

**Figure 8:**
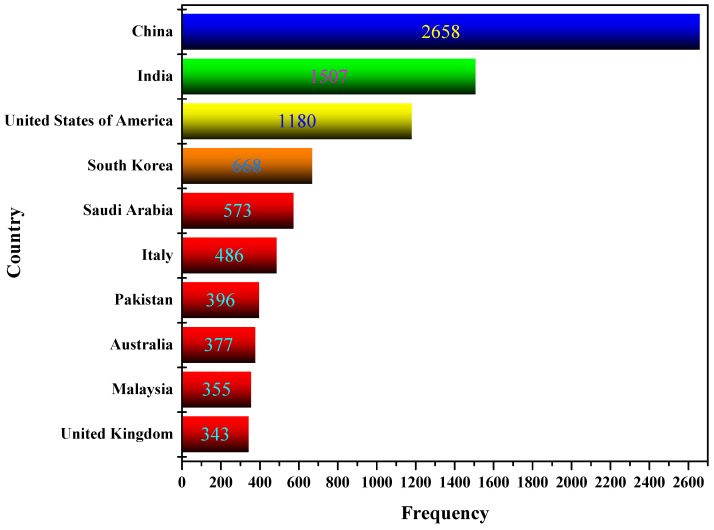
Scientific production by country.

Country Scientific Production quantifies the author’s appearances by country affiliations. This means that if an article contains three authors from the United States, Spain, and Italy, each country’s counter will increase by one. Therefore, each article will be counted as many times as there are authors (in the example above, three times). Consequently, the sum of the production indicator surpasses the total number of articles included in the dataset used for this study. As shown in Figure 8, China has produced more articles, followed by India and the USA.

**Figure 9:**
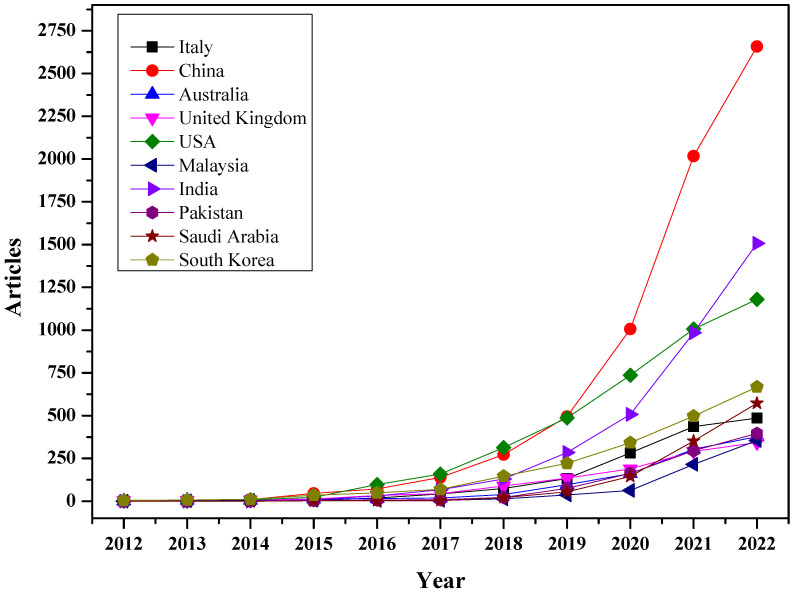
Production of top ten countries over time.

There is a direct relationship between total scientific production and countries’ production over time. Figure 9 shows the production of the top ten countries over time. It can be observed that the average production of countries was below 100 until 2016. A significant increase can be seen after 2017, especially in China, India, and the USA.

**Figure 10:**
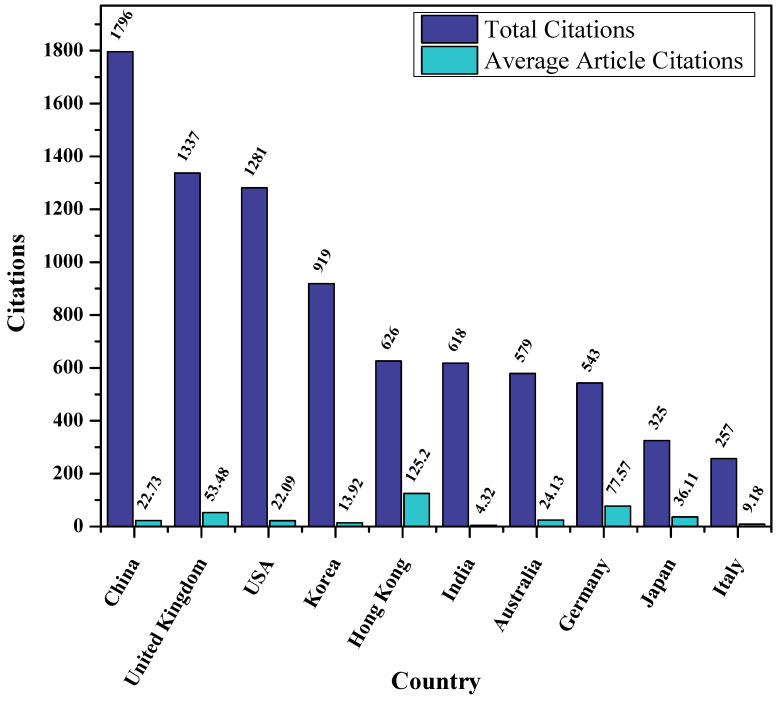
Total and Average articles citations of different countries.

Furthermore, a citation is used to describe the quality of the publication. As shown in Figure 10, China is the most cited country, with 1796 and 22.73 total and average citations, respectively. It is interesting to see that the United Kingdom follow China with 1337 and 53.48 total and average citations, though having fewer articles than all other top ten countries.

### 3.4. Keywords Analysis

**Figure 11:**
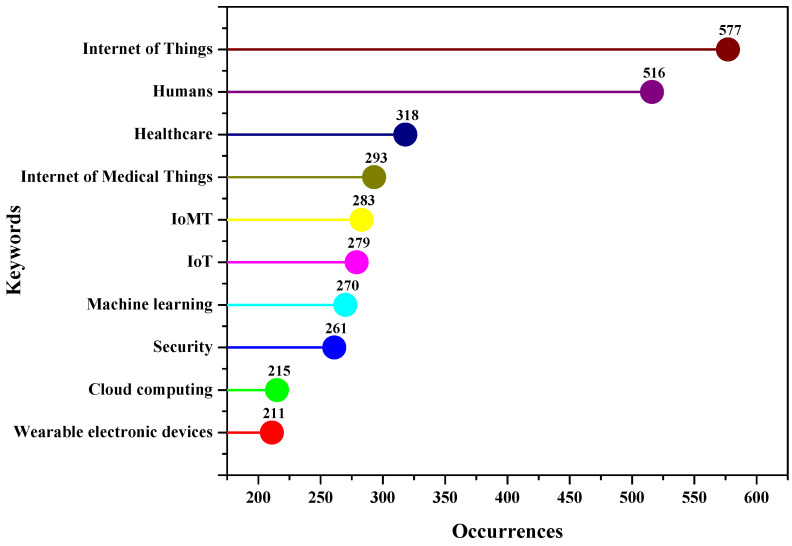
Most frequently used keywords in the dataset used for analysis.

Keywords, often known as search terms, are the words entered into the database’s search box to retrieve relevant documents. They represent the central ideas of any research topic and are the daily terms used to describe it. It may be difficult to locate the required documents without the proper keywords. Author keywords comprise a list of terms that authors believe best represent their research. The top 10 keywords used in the documents included in the dataset used for this study are shown in Figure 11. It can be noted that “internet of things” is a widely used term as it is the base for IoMT research. This is followed by the keywords “human” and “healthcare”. The terms “internet of medical things” and “IoMT” have been used 293 and 283 times, respectively. These results are also crucial because they represent the search terms used to build up the search string for document retrieval for this study. Author keywords are an effective tool for investigating the knowledge structure of any scientific field.

**Figure 12:**
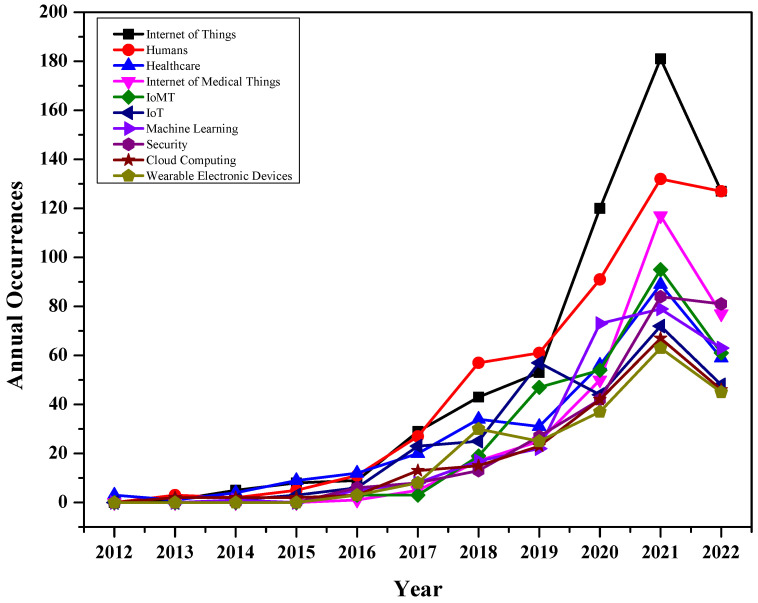
Annual Occurrences of author’s keywords.

The annual occurrence of keywords reflects the importance of any given topic over time. Figure 12 shows the trending keywords between 2012 and July 2022. As discussed, the term “Internet of Things” is widely used as the base for IoMT research. The figure indicates that all these terms were used less than 20 times from 2012 to 2016. There has been significant growth for all these terms after 2016. The terms “internet of medical things” and “IoMT” have grown exponentially in the last three years, indicating their importance. There are several factors and trends that caused the growth of IoMT market. These include the technological advancement in the healthcare sector in recent years. The growing partnership between university and healthcare sectors, as well as end-users, leads to the adaption of IoMT-based products. The significant growth in the past two years is due to COVID-19, which has had a positive impact on IoMT demand across all regions [43].

### 3.5. Knowledge Synthesis

For various reasons, drawing a big picture of scientific knowledge has always been desirable. Science mapping attempts to find representations of intellectual connections within the dynamically changing scientific knowledge system [44]. In simple words, science mapping aims to find scientific research’s structural and dynamic aspects using knowledge structure.

Science mapping allows users to investigate scientific knowledge from a statistical point of view.

#### 3.5.1. Conceptual Structure

**Figure 13:**
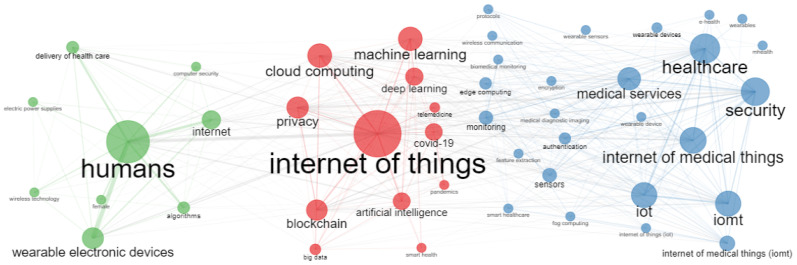
Co-occurrence Network.

The conceptual structure aims to target the main themes and trends. The co-occurrence network is created based on the Walktrap algorithm [45], and association is used as the normalisation method shown in Figure 13. The words that appear together in a document are related to a network. The conceptual structure is used to understand different topics of a research field and the associated issues. It also highlights the evolution of study over time. The Co-occurrence network in Figure 13 shows the evolution of the IoMT research. Each network node represents a keyword or study topic, and its size is proportional to its degree. It can be seen that there were a few keywords centred around “humans” with “algorithms”, “wearable electronic devices”, “wireless technology”, and “delivery of healthcare”. The second cluster is based on the Internet of Things, including “machine learning” and “deep learning”, “blockchain” and “cloud computing”, and “security” and “privacy” issues. Notably, “Internet of Things” is of key importance in this cluster due to its role as a network connector.

The third network merges the previous two and focuses on the Internet of Things for medical or healthcare. This network is more complex and dense, with a greater number of nodes and links, which suggests the diversity and increase of research topics. The six largest nodes are “healthcare”, “medical services”, “IoT”, “IoMT”, and “security”. The fact that the degree of “security” is in between the “healthcare” and “internet of medical things” indicates that security is a major issue with IoMT and is therefore receiving more recognition from scholars.

**Figure 14:**
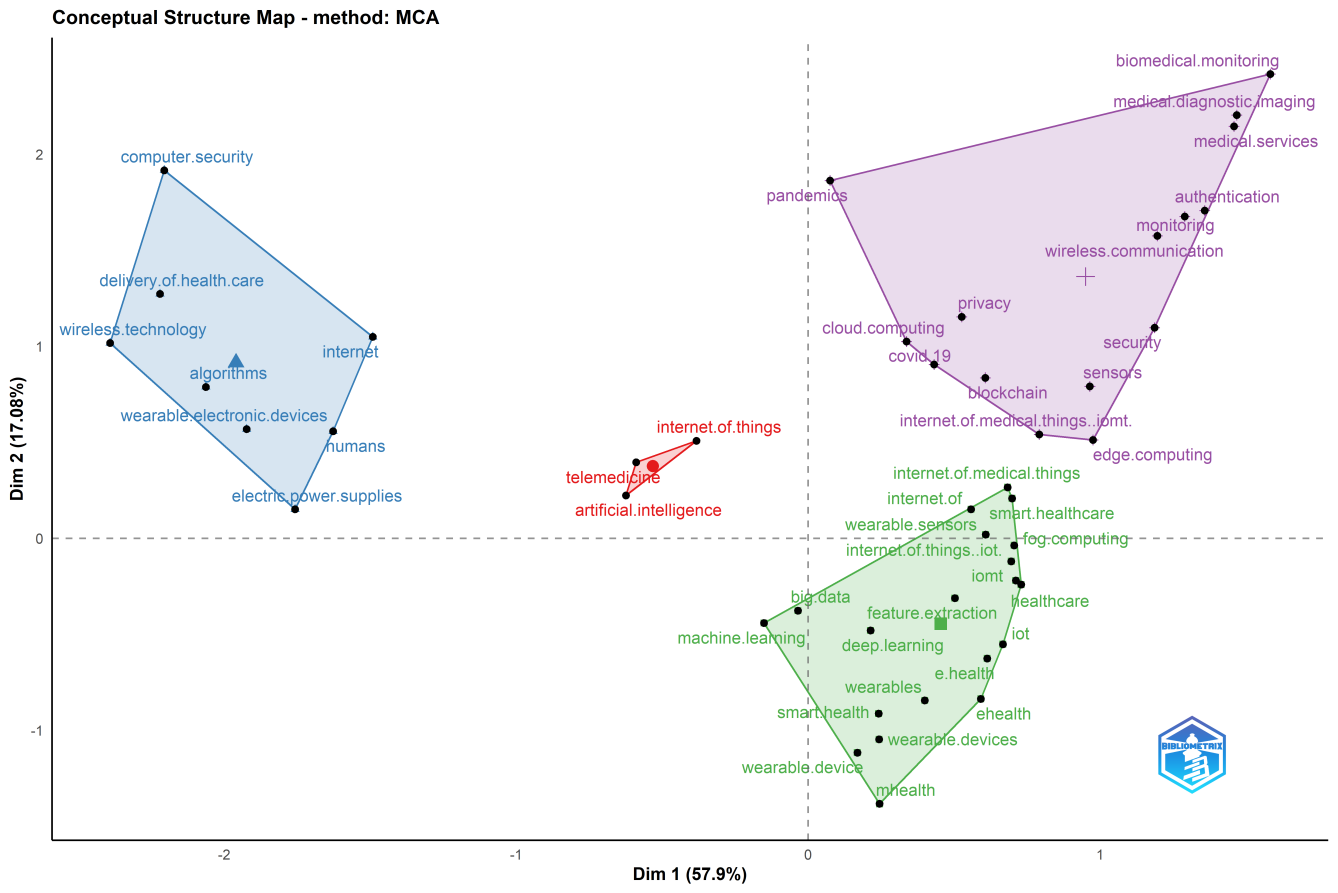
Factorial Analysis using the Biblioshiny package. Clusters are identified by hierarchical clustering.

To understand the subfields of a research area, factorial analysis is used. This is a data reduction technique, and different dimensional reduction techniques could be used, such as corresponding analysis, multiple corresponding analysis (MCA), and multidimensional scaling. The keywords are divided into four distinct areas highlighted in various colours. The proximity between words corresponds to the shared substance. These shared substances are created based on the keywords that are close or distant from each other. Keywords are close to each other only if many documents treat them together and vice versa. The factorial analysis based on MCA can be found in Figure 14. Each colour represents a cluster of words for a specific topic or subfield.

The factorial analysis identifies four dominating macro clusters in the chosen research subject. The first cluster (top left) consists of paper targeting algorithms, wireless technology, and the delivery of healthcare and wearable electronics devices. The second cluster (centre) brings together the internet of things, telemedicine and artificial intelligence. Cluster three (top right) and cluster four (bottom right) are the most dominant clusters and are linked together. Cluster three represents their search themes of remote monitoring of patients and privacy and security issues. The fourth cluster targets the use of wearable and smart health devices and the use of the machine and deep learning techniques for feature extraction.

#### 3.5.2. Social Structure

**Figure 15:**
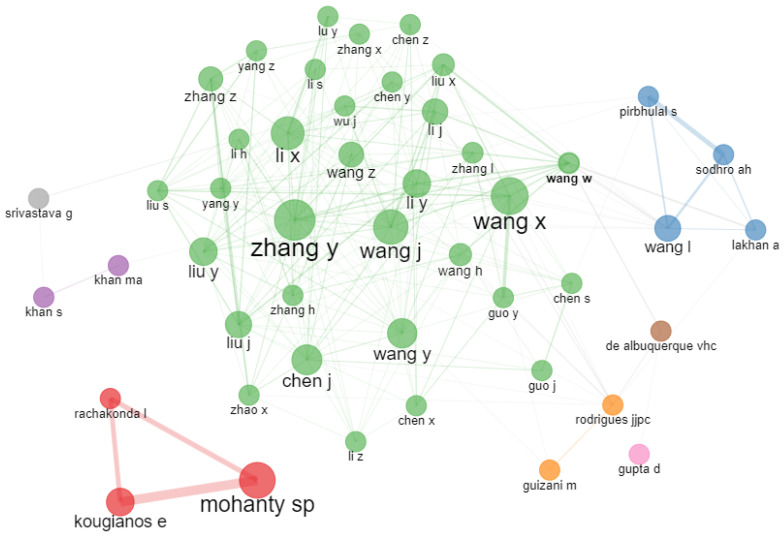
Co-authorship network using the bibliometric R package.

The social structure shows the relationship between different research entities, including authors or institutions. The most common form of social structure is the co-authorship network [46]. It is used to discover a regular and influential group of authors and relevant institutions in any research field. The co-authorship network for IoMT-related research is depicted in Figure 15.

The figure shows the clusters of authors that collaborate for the possibility of discovering new knowledge. Only a single big cluster can be seen in the network, which indicates that collaborations need to be increased to combine many types of knowledge and abilities to solve complicated health problems using IoMT.

**Figure 16:**
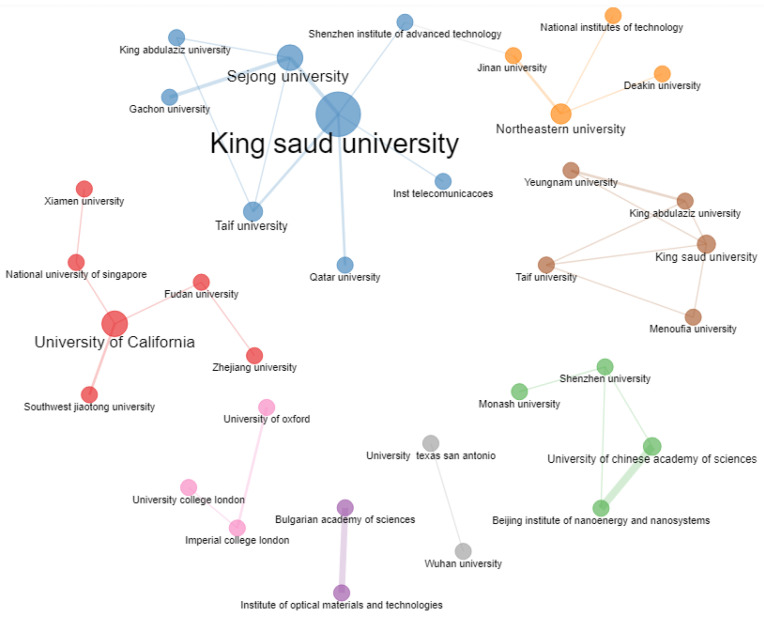
Collaboration network of different universities for IoMT research.

Research collaboration is an essential aspect of any research field nowadays. Figure 16 shows a collaboration network of different universities. There are eight different clusters in the network based on a minimum of two collaborating universities. The network shows that the research collaboration is limited to countries located in a specific region. It can also be seen that most of the China universities are collaborating throughout the world. The size of the bubble in the network shows the extent of collaboration. For example, Kind Saud University of Saudi Arabia collaborates with five other universities.

Based on the scientific collaboration among different institutions, it can be observed that collaboration within the same country/region is relatively high compared to those from different institutions and countries/regions.

**Figure 17:**
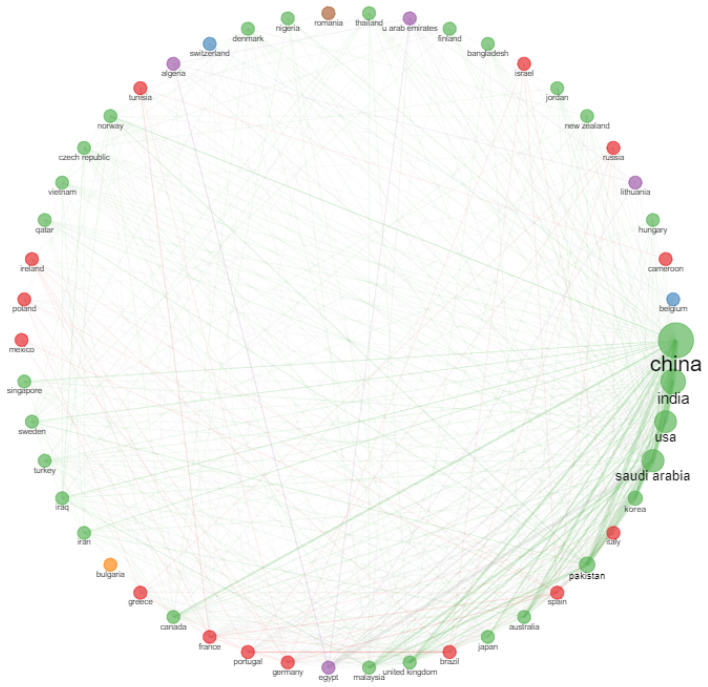
Collaboration network of different countries for IoMT research.

The collaboration network for different countries given in Figure 17 shows that China is the leading country in collaboration for IoMT research. This is followed by India, Saudi Arabia and the USA.

**Figure 18:**
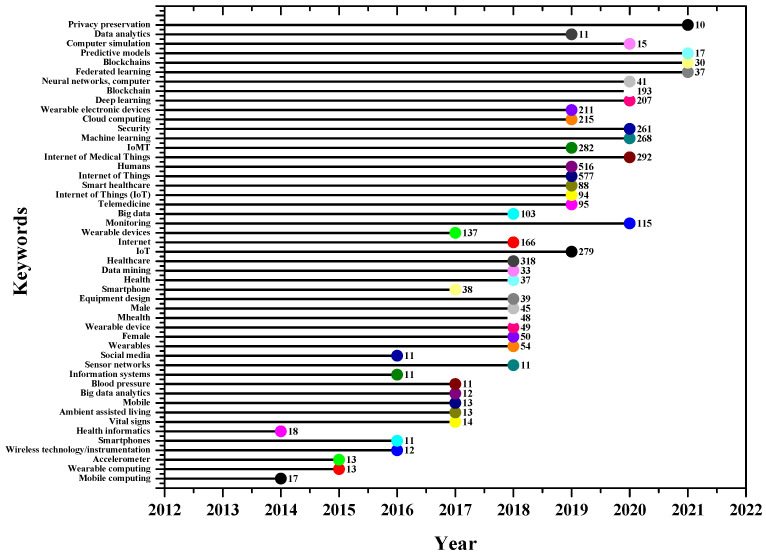
The trending topics; Minimum count was set to 10 each year.

The bibliometric analysis depicted research trends of IoMT-related research: (1) the growth rate of IoMT-related publications has grown rapidly over the past few years, and the rate has shown a trend of continuous growth; (2) high and middle-income countries are the main force of healthcare–related IoT research; (3) the majority of IoMT research was focused on activity recognition, monitoring, wearable devices, self-tracking, and mobile health; (4) data analytics, privacy, AI and blockchain, cloud, and edge computing are some of the recent trends in the IoMT related research as shown in Figure 18. For trending topics, the keyword count per year criteria is used. A minimum value of 10 was used to retrieve the documents where specific keywords were used.

The figure shows that privacy preservation, data analysis, blockchain, federated learning, and neural networks are trending topics in 2021 and 2022. This is not surprising, as the data obtained from the IoMT need to be processed using different machine learning and deep learning techniques. The keyword “privacy preservation” indicates the fact that privacy issues are currently being investigated together with the use of blockchain and cloud computing. The figure also indicates that IoMT became a hot topic of research in 2019 and 2020.

IoMT research is expanding rapidly, and possible applications have been demonstrated in various home health and patient-centred care settings. However, there are currently only a few instances where such strategies have been successfully implemented. Future IoMT-related research should focus on bridging the gap between healthcare-related IoMT research and clinical applications.

## 4. Open Research Areas

### 4.1. IoT for the Personalised Needs of the People

Although various IoMT devices are being developed, ranging from health monitoring and tracking, these IoMT devices and systems need to be tailored to the personalised needs of the individual. For example, IoMT-based tracking and monitoring systems for care homes should consider old age people [47]. These people do not prefer wearable devices; instead, unobtrusive devices must be used to get all the essential data.

### 4.2. Involvement of Real Users in the Development Process

A crucial factor that needs to be considered for any system design and development is the involvement of end users at the earliest stage of the project. The requirements must be collected from the end-users of the system. Furthermore, these users must be involved in the development throughout the project life cycle. Their feedback needs to be accommodated to enhance the user experience.

### 4.3. Cost-Effective Solutions Especially for the Less Developed Countries

Most studies do not consider the cost associated with these IoMT projects. For real-world and commercial IoMT projects, the components, implementation, and maintenance costs must be considered, especially for less developed countries. Moreover, research in this area is expected to increase in the coming years. Therefore, there is a need for enhanced collaboration between developed and developing countries.

### 4.4. The Need for Case Studies

Conceptualisations dominate most of the highly referenced and co-cited works reviewed in this study. A few applications of IoMT contain case studies, which would provide a more comprehensive understanding of this technology. Therefore, future research should also focus on case studies to better understand how IoMT originates, how it is used, and the benefits and problems associated with it.

### 4.5. Security and Privacy Issues

Compared to the Internet of Things, the IoMT project required more security, privacy, and interoperability [31]. The security dimension encompasses confidentiality, access control, data protection, and encryption concerns. These topics must be further explored with application-specific issues [48]. The privacy of health data is an important area of research due to its impact on the success of IoMT adoption. Failure to protect data confidentiality and integrity and manage risks associated with unauthorised access may result in the inability to realise the benefits of IoMT [49].

## 5. Limitations of the Study

For this study, the authors tried their best to search comprehensively for all the material related to the topic under discussion. However, this study still has many limitations.

- Relevant studies are only searched for the period 2012–2022.
- Although seven major databases are used for searching relevant studies, there are still many un-indexed journals and publications from such journals might have been missed.
- No search string can be 100% perfect; therefore, false positive and false negative results are always possible.
- Only English language papers are considered for the study, which may have biased results towards English-speaking countries to some extent.

## 6. Conclusions

This paper presents an integrated methodology to perform bibliometric analysis. The proposed methodology is discussed step by step. The method integrates data from seven major databases using different tools to avoid manual work. Furthermore, the proposed methodology could be used to conduct all-inclusive bibliometric studies on any topic. The proposed methodology is evaluated by performing a bibliometric analysis of the Internet of Medical Things. The bibliometric analysis provides a detailed review of IoMT-related research for the last ten years. This paper also highlights the open research area in the IoMT research that needs further investigation. This study’s findings and recommendations for further research will benefit academics, policymakers, and healthcare practitioners who seek to collaborate in these fields in the future.

The proposed methodology does not require any coding skills. The user does not have to be an expert in R Studio usage beyond the Bibliometrix package required for bibliometric analyses. Additionally, it does not involve the use of shareware software, making it potentially accessible to all academics, including those from developing countries. The proposed methodology is, however, susceptible to human error, as is the case with every manual step of the proposed method. However, the potential advantages of being user-friendly and having the ability to analyse seven major databases outweigh this limitation. A major concern that needs further investigation is how to handle citation metadata when integrating datasets from multiple sources. These databases, such as Scopus and WoS, are closed systems that only count citations from publications within the same database. The number of citations for the same record may vary depending on the data source. This paper considered papers with the highest number of citations while eliminating duplicates. It is further recommended that researchers use the cross-validation procedure to determine the provenance of a database.

## Figures and Tables

**Table 1:** Databases searched along with the search strings, retrieved results, and data format for each database. The GS string is divided into two strings because the Publish or Perish software does not allow downloading more than 100 results.

Database	Search Terms	Results	Data Format
Scopus	“Internet of medical things” OR “IoMT” OR “Internet of Health Things” OR “IoHT” OR “Internet of Things for Medical Devices” OR “IoT Medical Devices” OR “Smart Health” OR “Wearable Devices” AND “healthcare” AND “Medical”.	1561 Results	.bib
Google Scholar	1. healthcare “internet of medical things” OR IoMT OR “Internet of Health Things” OR IoHT OR “Internet of Things for Medical Devices” OR “IoT Medical Devices” OR “Smart Health” OR “Wearable Devices”.	1. 408 Results	Exported to Excel from Perish or Publish software
	2. medical “internet of medical things” OR IoMT OR “Internet of Health Things” OR IoHT OR “Internet of Things for Medical Devices” OR “IoT Medical Devices” OR “Smart Health” OR “Wearable Devices”.	2. 958 Results	
		Total: 1045	
Web of Science	“Internet of medical things” OR IoMT OR “Internet of Health Things” OR IoHT OR “Internet of Things for Medical Devices” OR “IoT Medical Devices”	997 Results	.bib
IEEE Explore	“Internet of medical things” OR “IoMT” OR “Internet of Things for Medical Devices” OR “IoT Medical Devices” OR “Internet of Health Things” OR “IoHT”.	788 Results	.bib
ACM	“Internet of medical things” OR “IoMT” OR “Internet of Health Things” OR “IoHT” OR “Internet of Things for Medical Devices” OR “IoT Medical Devices” OR “Smart Health” OR “Wearable Devices” AND “healthcare” AND “Medical”.	985 Results	.bib
Science Direct:	“Internet of medical things” OR “IoMT” OR “Internet of Things for Medical Devices” OR “IoT Medical Devices” OR “Internet of Health Things” OR “IoHT”.	120 results	.bib
Pubmed:	“Internet of medical things” OR “IoMT” OR “Internet of Things for Medical Devices” OR “IoT Medical Devices” OR “Internet of Health Things” OR “IoHT”.	926 results	.txt
Total Results		6422 Results	.xlsx
Not in the English Language		Eliminated while downloading	.xlsx
Eliminate Duplicates and Irrelevant		1184	.xlsx
Final dataset		5238	.xlsx

**Table 2:** Overview of the final dataset used for bibliometric analysis.

Description	Results
Timespan	2012:2022 (July 2022)
Sources (Journals, Books, etc.)	2365
Documents	5304
Annual Growth Rate %	40.92
Document Average Age	2.2
Average citations per doc	6.57
Keywords Plus (ID)	7796
Author’s Keywords (DE)	9527
Authors	14,404
Authors of single-authored docs	381
Single-authored docs	439
Co-Authors per Doc	4.06
International co-authorships %	14.4

**Table 3:** Average Citation Per Year, TC: total citations.

Year	No of Articles	Mean TC per Art	Mean TC per Year	Citable Years
2012	13	0.00	0.00	10
2013	14	0.21	0.02	9
2014	34	0.53	0.07	8
2015	48	2.25	0.32	7
2016	112	3.43	0.57	6
2017	199	4.78	0.96	5
2018	327	24.80	6.20	4
2019	504	11.53	3.84	3
2020	838	14.80	7.40	2
2021	1195	0.38	0.38	1
2022 (July)	807	0.00	NA	0

**Table 4:** Top 10 Journals with *h*, *g* and *m* index as well as total citation counts based on all seven databases.

Element	*h*_index	*g*_index	*m*_index	TC
IEEE Access	29	47	4.143	2778
IEEE Internet Of Things Journal	22	37	3.667	1480
Future Generation Computer Systems	17	25	3.4	634
Computer Communications	11	20	2.75	431
Sensors	11	20	1.833	508
Sensors (Switzerland)	10	14	1.111	367
Electronics	8	15	2.667	245
IEEE Sensors Journal	8	19	0.727	465
IEEE Transaction on Consumer Electronics	8	10	1.6	181
IEEE Journal of Biomedical and Health Informatics	7	13 -	1.167	170

## Data Availability

The data presented in this study are available on request from the corresponding author.

## Abbreviations

The following abbreviations are used in this manuscript:

IoMT	Internet of Medical Things
IoT	Internet of Things
WoS	Web of Science
IoHT	Internet of Health Things
AU	Author
TI	Title
PY	Publication Year
TC	Total Citations
GS	Google Scholar
MCP	Multiple Country Publications
SCP	Single Country Publications
MCA	Multiple Corresponding Analysis

## Appendix A. Advanced Google Scholar Search

1. To access the advanced search option on Google Scholar, click on the three-line icon in the upper left corner of the search page.
2. Click on Advanced search.
3. From the Advanced search pop-up box, use “in the title of the article” to limit the search to the document title only.

## Appendix B. Integrating Data from Multiple Sources Using R

library (RefManageR)

> scopus = convert2df(file. choose(), dbsource = “scopus”, format = “bibtex”)

> wos = convert2df(file. choose(), dbsource = “wos”, format = “bibtex”)

> pubmed = convert2df(file. choose(), dbsource = “pubmed”, format = “txt”)

> Coverteddata = as.data.frame(mergedSources)

For IEEE > IEEE = convert2df(file.choose(), dbsource = “wos”, format = “bibtex”)

Science Direct: > ScienceDirect = convert2df(file.choose(), dbsource = “wos”, format = “bibtex”)

> > mergedSources = mergeDbSources(ACM, IEEE, pubmed, ScienceDirect

,scopus, wos, remove.duplicated = TRUE)

> writexlsx(Coverteddata, ”Path..”)

## Appendix C. Integrating Data from Multiple Sources Using Excel

1. Open a new document
2. Click on data and then Get data, and then “Launch Power Query Editor”.
3. On the top right, click on ‘New Source’-> file ->Excel Workbook and Open the first file.
4. Import all the remaining files similarly.
5. After inserting all the files, select anyone and then click Append Queries -> Append Queries as new, and the following window will appear.
6. In the new window, select “Three or more tables and add all the sheets to the “Tables to append” window (Hold ctrl to select more than one sheet while adding) and click OK.
7. This will append all the sheets together and give them a name such as “Append”. Click on Close and Load, which will load the data from all sheets to a new sheet.
8. Save the data in .xlsx format.
